# Case Report: Severe thrombocytopenia induced by adalimumab in rheumatoid arthritis: A case report and literature review

**DOI:** 10.3389/fphar.2022.1041884

**Published:** 2022-10-25

**Authors:** Tiantian Liao, Mengqing Li, Tian Yuan, Qifu Hong, Yu Zeng, Dan Yu, Qiong Yu, Limei Yu, Tao Pu

**Affiliations:** ^1^ Department of Nephrology, The Affiliated Hospital of Zunyi Medical University, Zunyi, China; ^2^ Key Laboratory of Cell Engineering, The Affiliated Hospital of Zunyi Medical University, Zunyi, China; ^3^ The Team of Scientific and Technological Innovation Talents on The Basic and Clinical Research of Amniotic Membrane and Bone Marrow Stem Cells, Zunyi, China

**Keywords:** rheumatoid arthritis, adalimumab, thrombocytopenia, complication, biologic therapy

## Abstract

Rheumatoid arthritis (RA) is a chronic inflammatory disease characterized by persistent joint inflammation. In recent decades, biological agents such as anti-tumor necrosis factor-α (TNF-α) drugs have been applied in the treatment of RA and it achieved great improvement. The treatment has its side effects, but severe thrombocytopenia is very rare. In this case report we described the occurrence of severe thrombocytopenia in a patient with RA who was treated with adalimumab. Specially, the symptoms of the RA are not significantly improved by adalimumab treatment and severe thrombocytopenia it induced is resistant to treatment. After receiving four doses of adalimumab, the patient’s platelet count dropped to 4 × 10^3^/μl. We halted adalimumab and administered glucocorticoids, interleukins, and platelet transfusion. On the sixth day, the platelet count rose to 52 × 10^3^/μl. Lab tests and bone marrow pictures were unremarkable. Patient was treated with prednisone for maintenance. On day 17, the platelet count declined to 12 × 10^3^/μl. We started the patient on methylprednisolone and recombinant human thrombopoietin (rh-TPO), but the effect was not significant. On day 25, intravenous immune globulin (IVIG) was applied in place of the rh-TPO. On 29th day, the patient’s platelets returned to normal. We summarized the existing literature on thrombocytopenia induced by anti-TNF-α drugs. This case suggested immunoglobulins could be considered for the treatment of refractory thrombocytopenia.

## Introduction

Rheumatoid arthritis is a chronic systemic autoimmune illness that can cause joint discomfort, swelling and deformity. It is characterized by chronic synovial inflammatory reaction. The main pathological manifestations include synovial lining cell proliferation, interstitial inflammatory cell infiltration, microvascular neogenesis, pannus formation and cartilage and bone tissue destruction. It seriously affects the quality of life of the patients and multiple systems of the body. There are many molecular mechanisms in rheumatoid arthritis, such as the IL31/IL33 axis, which leads to gene and protein activation of inflammatory diseases through cascade reactions (Murdaca et al., 2019). Subsequently involved in the secretion of TNF-α. Tumor necrosis factor is one of the major inflammatory cytokines in the RA patients. It regulates the production of inflammatory factor like IL-6, IL-8, MCP-1, and VEGF, as well as the recruitment of immune and inflammatory cells to the affected joint (Lim et al., 2018). Therefore, it plays an important role in the pathological development of RA. As a therapeutic target in rheumatoid arthritis, anti-TNF-α drugs have been used in the RA patients since mid-1990s. Numerous clinical studies have demonstrated that anti-TNF-α drugs can improve not only the clinical signs and symptoms of RA patients, but also their joint function and imaging results (Caporali et al., 2018). Anti-TNF-α drugs were effective and were well tolerated by many RA patients. Multiple recommendations advocate the clinical use of anti-TNF-α drugs. 2021 American College of Rheumatology Guidelines recommend that patients who do not respond adequately to methotrexate monotherapy be considered for the addition of anti-TNF-α drugs (Fraenkel et al., 2021). In addition to RA, anti-TNF-α drugs are also used in the treatment of multiple autoimmune diseases, such as Crohn’s disease, ulcerative colitis, psoriatic arthritis, etc. (Lim et al., 2018). However, like other biologics, the incidence of anti-TNF-α drugs adverse effects is increasing as their clinical usage becomes more prevalent. Infections, including tuberculosis relapse and other opportunistic infections are the most serious side effects of anti-TNF-α drugs therapy. Other side effects include infusion reactions, rash, systemic symptoms, demyelinating disease, exacerbation of congestive heart failure, lupus-like autoimmune disease, liver disease and hematologic abnormalities (Bessissow et al., 2012). The common hematological complications are neutrophil decrease and thrombocytopenia (Dogra and Khullar, 2013). However, these side effects are short-lived and frequently fade away once you stop using the drug. Severe and persistent thrombocytopenia is a very rare complication and has only been reported in few individual cases.

## Case presentations

**FIGURE 1:**
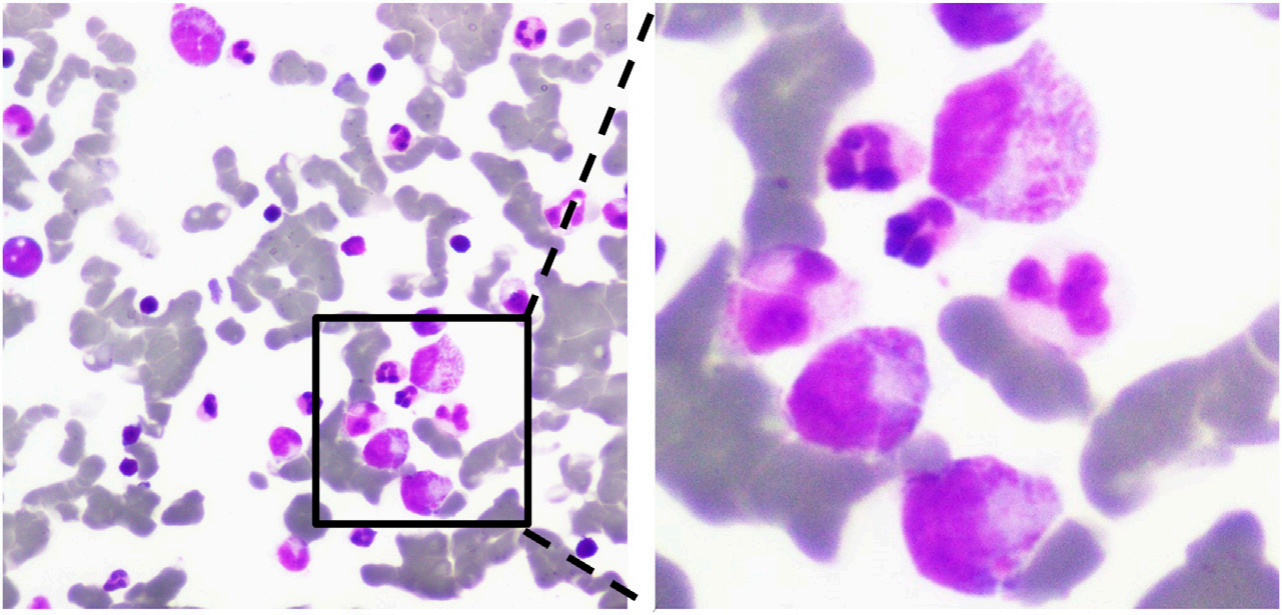
Morphological examination of bone marrow cells. Megakaryocytes proliferate actively. The ratio of platelet- producing megakaryocyte was decreased, and platelets were few (×100, ×400).

A 53-year-old male presented to the outpatient department with systemic bleeding spots and oral mucosal blood blisters. The patient was diagnosed with rheumatoid arthritis for 20 years and followed-up in the outpatient clinic regularly. He was treated with methotrexate (MTX) at a dose of 12.5 mg/week, and combined with hydroxychloroquine, leflunomide, and prednisone intermittently. Three months ago, adalimumab was given biweekly at a dosage of 40 mg due to the disease’s poor management. Leflunomide and hydroxychloroquine were stopped. Unfortunately, the patient’s symptoms did not improve after receiving adalimumab treatment. He had no significant medical history other than RA. And platelet counts before treatment with adalimumab were between 150 × 10^3^/μl and 300 × 10^3^/μl. After admission, laboratory revealed a platelet count level of 4 × 10^3^/μl, a WBC count of 15.96 × 10^9^/L and a hemoglobin count of 135 g/L. Besides, rheumatoid factor and anti-cyclic citrullinated peptide antibody were 814 IU/ml and 177.70 U/ml, respectively. The laboratory data revealed that the levels of liver transaminases, creatinine, and estimated glomerular filtration rate (eGFR) were normal. Immunological tests for immunoglobulin, anti-double-stranded DNA antibody, anti-nucleosome antibody, and anti-SM antibody were negative. Serological screening for cytomegalovirus, herpes simplex virus, HIV and hepatitis B were negative. Physical examination revealed normal. After hospitalization, adalimumab and MTX were stopped, dexamethasone 10 mg and interleukin-11 (IL-11) 3 mg, were given immediately. Three units of apheresis platelets were transfused. On the third day of hospitalization, the platelet count rose to 16 × 10^3^/μl. The bone marrow puncture was performed and bone marrow smear showed megakaryocytes was actively proliferating and no malignant lesions (Figure 1). The treatment of the combination of corticosteroids and IL-11 was continued. Since the platelet count was still below 20 × 10^3^/μl, three units of apheresis platelets were given again. On the sixth day, the platelet count rose to 52 × 10^3^/μl, the patient was discharged and prednisone (40 mg, once a day) were applied for maintenance.

**FIGURE 2:**
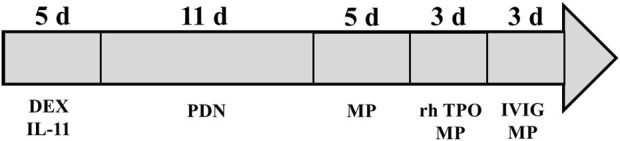
A case of adalimumab-induced severe thrombocytopenia with treatment process. DEX, dexamethasone; PDN, prednisone; MP, methylprednisolone; rh-TPO, recombinant human thrombopoietin; IVIG, intravenous immune globulin.

**FIGURE 3:**
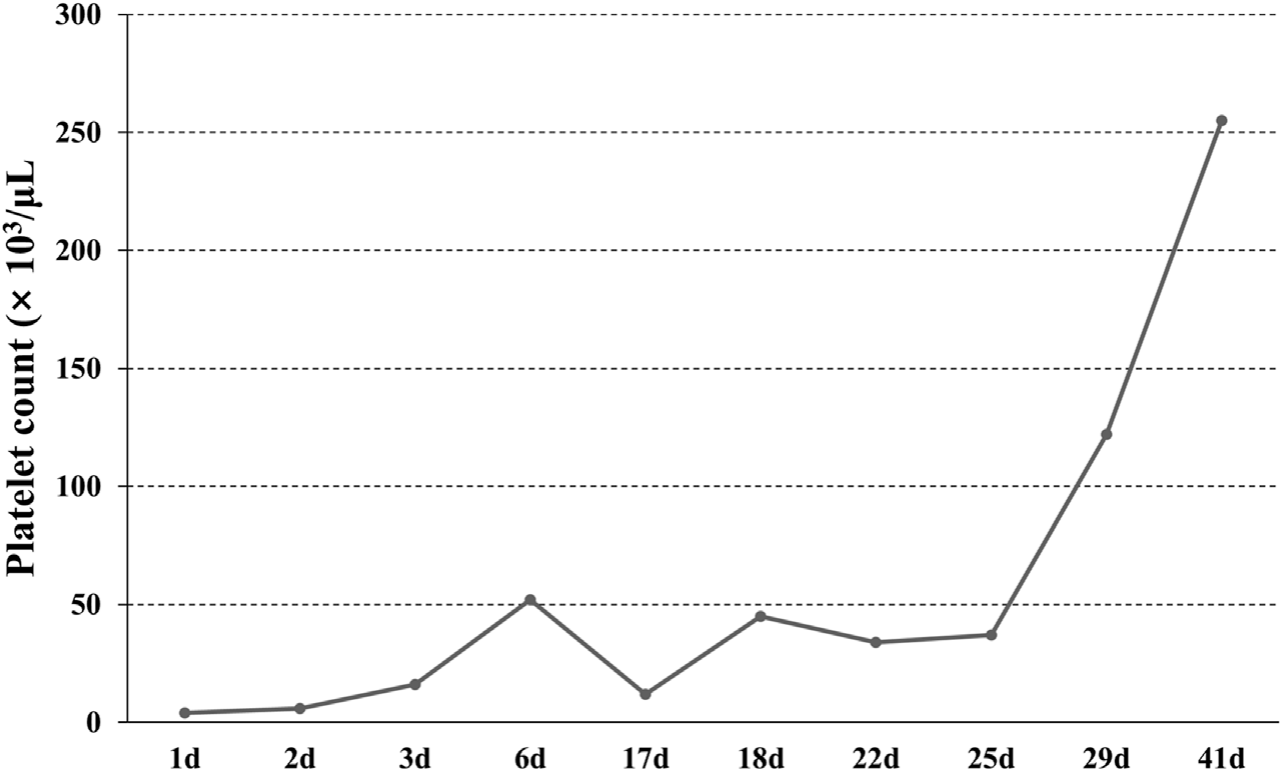
The change of platelet count. The patient was admitted with severe thrombocytopenia on the first day, and that the platelet count returned to normal after 29 days of continuous treatment.

The patient was readmitted for recurrent severe thrombocytopenia 11 days after discharge. Laboratory revealed a platelet count level of 12 × 10^3^/μl, and coagulation tests were normal. Anti-platelet antibody and anti-lipid coagulation antibody were negative. After admission, methylprednisolone (40 mg, once a day) was used for therapy. On the fifth day of hospitalization, rh-TPO (1 mg, once a day) was applied when the platelet count rose to 34 × 10^3^/μl. Three days later, laboratory revealed a platelet count level of 37 × 10^3^/μl. IVIG was applied in place of the rh-TPO, 15 g on the first day, 10 g on the second and third days. On the twelfth day, rechecking platelet count revealed a level of 122 × 10^3^/μl. Treatment process is shown in (Figure 2). The patient was discharged. The patient received oral prednisone 30 mg once a day for maintenance. He tapered off his oral glucocorticoids and finally discontinued. The patient was not on anti-TNF-α drugs after discharge, and methotrexate 7.5 mg once a week, leflunomide 20 mg twice a day and hydroxychloroquine 200 mg twice a day were given. He took prednisone tablets according to the condition. Joint pain still haunted the patient’s lives. Then, his treatment regimen was modified to methotrexate 12.5 mg once weekly and baritinib 2 mg once daily. Until this case report was written, the patient’s condition is well controlled. Blood routine examination showed that platelet count increased to normal level and maintained (Figure 3).

## Discussion

As a multisystem autoimmune disease, RA can not only lead to systemic joint dysfunction, but also implicate other organ and system, such as the heart, lungs, kidneys, and blood system. Among RA patients, active synovitis, bone, and cartilage destruction are an important stimulating factor for platelets. In the active stage, RA patients may have a relatively high platelet count, but it would return to normal levels in the remission state (Yazici et al., 2010). However, thrombocytopenia in RA patients is unusual and often related to the side effects of anti-rheumatic drugs or multiple drug interactions. Thrombocytopenia caused by MTX or other disease-modifying medications has been reported widely (Wang et al., 2018). Besides, there are few reports about the RA patients complicated with thrombocytopenia caused by primary blood disorders such as immune thrombocytopenic purpura (ITP) or thrombotic thrombocytopenic purpura (TTP) (Bowman, 2002). The patient had no fever or neurological signs, and his laboratory tests showed WBC, RBC, LDH, creatinine, and eGFR in the normal range. The antiplatelet antibody testing was negative. We performed the bone marrow examination. The results showed no malignant lesions. The causes of thrombocytopenia were aplastic anemia (AA), TTP and ITP, which were ruled out (Akin and Haznedaroglu, 2021).

It has been widely reported that disease-modifying anti-rheumatic drugs (DMARDs) could induce cytopenia, specially MTX. MTX could block dihydrofolate reductase, inhibit the production of nucleic acids, and lead to hematologic toxicity. MTX-induced hematological complications include leukopenia, anemia, thrombocytopenia, and pancytopenia. However, a meta-analysis based on multiple randomized controlled trials found that the incidence of cytopenia was significantly reduced since RA patients took low-dose MTX (7.5–25 mg, weekly) with folic acid supplementation. Thrombocytopenia is a less common complication which occurred in less than 1% RA patients (Vanni et al., 2020). Our patient was treated with low-dose MTX (7.5–25 mg, weekly) for years and follow-up regularly. Blood system abnormalities never occurred until adalimumab was administered. The patient took no other DMARDs during treatment with adalimumab. The bone marrow examination also found no abnormalities. The possibility of MTX or other DMARDs causing severe thrombocytopenia could be excluded. Therefore, adalimumab, a kind of anti-TNF-α drug, is likely to be cause of thrombocytopenia in this patient.

Mechanisms by which drug induced thrombocytopenia could be divided into immune-mediated and non-immune-mediated (Kam and Alexander, 2014). The formation of immune-mediated antibody leads to platelet destruction. The malfunction of megakaryocytes in the bone marrow is one of the non-immune-mediated processes. The bone marrow examination of the case revealed that the megakaryocytes were functioning normally. The patient’s platelet count returned to normal after IVIG therapy, supporting thrombocytopenia caused by immune-mediated mechanisms. According to different preparations, anti-TNF-α drugs can be divided into five groups: infliximab, adalimumab, etanercept, golimumab and certolizumab pegol. Infliximab commonly is a chimeric human-mouse monoclonal antibody and frequently utilized in clinical practice. Adalimumab and golimumab are fully human anti-TNF-α monoclonal antibodies (IgG1). Etanercept is a fusion protein consisting of human TNF receptor 2 attached to a human IgG1 Fc tail. Certolizumab pegol consists of a humanized Fab’ fragment bound to polyethylene glycol. As a monoclonal antibody and protein product, anti-TNF-α drugs have immunogenicity and could trigger an immunological response *in vivo* that results in the production of anti-drug antibodies (ADA). ADA may cause substantial side effects including infusion/allergic responses, thrombotic events, lupus-like events, vasculitis-like events, and drug-effectiveness reduction, etc. (Jani et al., 2018). Compared with murine chimeric antibodies, fully human antibodies have a lower risk of triggering immunological responses in humans, but they still have the potential to do so. Thrombocytopenia may be associated with ADA. However, the exact mechanism is still unclear (Jani et al., 2018). Current theories implied that a T cell-dependent pathway is primarily responsible for production of ADA. Monoclonal antibodies as antigens are presented to T-cells by antigen-presenting cells through the homologous interaction of MHC class II molecules and T-cell receptors. T helper cells then differentiate into Th1 and Th2 phenotypes. They interact with B cells to induce proliferation of plasma cells capable of producing ADA (Vaisman-Mentesh et al., 2020). The giant anti-TNF-α-ADA complex, which is typically found in multimers such as tetramers and hexamers may contribute to the adverse events. Because the complex is circular and the FC tail faces outward, the composite is so polymeric and irregularly shaped that the FC tail segments are near one another. Then tail of FC forms an internal complex, which facilitates the binding of complement C1 and activates the complement cascade (Atiqi et al., 2020). FcgR blockade/inhibition, complement inhibition, and possible T-cell regulation/induction of regulatory T-cells are the key hypothesized mechanisms of immunoglobulin. Probably because of this, immunoglobulin is effective.

Overall adverse event rates associated with anti-TNF-α drugs have been reported to be approximately 53%. Hematology-related complications such as neutropenia occur in less than 1% of patients. Isolated severe thrombocytopenia secondary to anti-TNF-α drugs has only been reported in individual case (Murdaca et al., 2009; Bessissow et al., 2012). Including the case reported this time, we summarized 20 cases of thrombocytopenia induced by anti-TNF-α drug (male/female = 8/12; mean age 49.25 years; median age: 50.05 years; age range: 15–75 years) (Table 1). The time from drug exposure to thrombocytopenia was 4 days–29 months (mean time: 29 weeks; median time: 19 weeks). Time to platelet recovery ranged from 6 days to 32 weeks (mean time: 10 weeks; median time: 7 weeks). Platelets returned to normal in 9 of the 20 patients after drug withdrawal (mean platelets 54.7 × 10^3^/μl; median platelets: 42 × 10^3^/μl; range: 10–128 × 10^3^/μl). The other 11 patients received other treatments, such as glucocorticoid (10/11), IVIG (4/11), double filtration plasmapheresis (DEPP) (1/11), rituximab (RTX) (1/11), rh-TPO (1/11), etc. Seven patients were re-treated with anti-TNF-α drug after their platelets returned to normal. However, four of the seven patients had a second decrease in platelets. Immunological tests were performed in eight individuals and found six of them were antinuclear antibody positive and four of them were antiplatelet antibody positive. Six individuals experienced other adverse effects. Including four cases of granulocytopenia and one case of erythema multiforme exudativum and pneumonia respectively.

**TABLE 1:** Summary case reports of severe thrombocytopenia after treatment with anti-TNF-α drugs.

Year	Author	Disease	Age/Sex	First/Rechallenge agents	BPC (×10^3^/ul)	Onset of thrombocytopenia	Serology	Other AEs	Treatment	Recovery time
2003	Vidal F (Vidal et al., 2003)	RA	60/F	IFX	<10	7 w	Neg	neutropenia	Stop IFX + GM-CSF	10 d
2004	Selby LA (Selby et al., 2004)	CD	15/M	IFX	4	6 d	antiplatelet Ab	NO	Stop IFX + GC + IVIG	6 d
2006	Pathare SK (Pathare et al., 2006)	RA	44/F	ETN/IFX one dose/ADA	38	6 w/N	Neg	NO	Stop ETN/Replace Anti-TNF-α drug	No data
		RA	56/F	IFX/ETN	26	29 m/N	ANA (1:160) dsDNA (>200 IU/ml)	NO	Stop IFX/Replace Anti-TNF-α drug	1 m
2007	Salar A (Salar et al., 2007)	CD	42/F	IFX/ADA	44	30 w/1 w	antiplatelet Ab	NO	Stop IFX + GC/Stop ADA	5 m/No data
2007	Hamaguchi M (Hamaguchi et al., 2007)	SSc overlap/RA	47/F	IFX	123	13 m	LAC, IgMACA (12.0 U/ml), ANA (1:320)	fever, pruritus, EME	Stop IFX + GC, anticoagulant	4 m
2009	Brunasso (Brunasso and Massone, 2009)	PSO	53/M	ETN/ETN	44	9 w/1 w	ANA (1:640)	NO	Stop ETN/Stop ETN	2 w
		PSA	67/F	IFX/ETN	1	29 w/17 w	Neg	NO	Stop IFX + GC/Stop ETN + GC	10 w/2 w
		PSO	55/M	IFX	18	30 w	ANA (1:1,064) antiplatelet Ab	NO	Stop IFX + GC + MMF + RTX	No recovery
		PSO	41/F	IFX	42	30 w	ANA (1:1,280) antiplatelet Ab	NO	Stop IFX	22 w
2011	Azevedo VF (Azevedo et al., 2012)	RA	54/F	ETN	60	4 m	No data	leucopenia	Stop ETN	1 m
		PSO	48/M	ETN	110	8 m	No data	leucopenia	Stop ETN	8 m
2012	Casanova MJ (Casanova et al., 2012)	CD	71/F	ADA/ADA	44	4 m/3 m	Neg	Pneumonia	Stop ADA + Stop AZA/Stop ADA + IVIG + GC were ineffective, added UST	No data
2013	Mocciaro F (Mocciaro et al., 2013)	CD	75/M	IFX/ADA	35	22 w/N	No data	No	Stop IFX/Replace Anti-TNF-α drug	1 m
2015	Matsumoto S (Matsumoto and Mashima, 2016)	UC	30/M	IFX	23	10 d	No data	No	Stop IFX ineffective, added GC	12 w
2016	Gomez G (Gomez et al., 2016)	UC	34/F	IFX	128	19 d	No data	Severe neutropenia	Stop IFX + antibiotics + G-CSF	No data
2016	Nagai Y (Nagai et al., 2016)	RA	68/F	IFX	2	4 d	ANA (1:320)	No	Stop IFX + GC ineffective, performed DEPP	14 d
2018	NAKAHARA T (Nakahara et al., 2019)	PSO	43/F	ADA	42	18 m	No data	No	Switching to SEC ineffective. Then stop SEC. Platelet count recovery, switching to UST. Platelet count decreased slightly. Then stop UST	4 m
2021	Al-Tkrit A (Al-Tkrit et al., 2021)	HS	29/M	ADA	1	2 y	No data	No	Stop ADA + IVIG + GC	No data
2022		RA	53/M	ADA	4	3 m	Neg	No	Stop ADA + GC + IL-11 ineffective, GC + rh TPO ineffective, Switching to GC + IVIG	28 d

The table is modified and updated based on the tables published by Brunasso and Massone (2009), Casanova et al. (2012), and Nagai et al. (2016). BPC, blood platelet count; N, normal; F, female; M, male; AEs, Adverse events; Neg, negative; RA, rheumatoid arthritis; CD, Crohn’s disease; UC, ulcerative colitis; SSc, Systemic sclerosis; HS, hidradenitis suppurativa; PSA, psoriatic arthritis; PSO, psoriasis; IFX, infliximab; ETN, etanercept; ADA, adalimumab; AZA, azathioprine; GM-CSF, Granulocyte-macrophage colony-stimulating factor; G-CSF, Granulocyte colony-stimulating factor; IVIG, intravenous immune globulin; GC, glucocorticoid; RTX, rituximab; MMF, mycophenolate mofetil; UST, ustekinumab; DFPP, double filtration plasmapheresis; SEC, secukinumab.

In addition to platelet transfusion, the patient was repeatedly treated with glucocorticoids, rh-TPO, interleukin-11, and other medicines after developing severe thrombocytopenia. The patient’s platelet count rose to a save range for a period, but severe thrombocytopenia recurred despite glucocorticoid maintenance. Platelets returned to normal following IVIG treatment, and remained stable during subsequent long-term follow-up. This example demonstrates that IVIG might be superior to glucocorticoids in patients with anti-TNF-α drugs induced refractory thrombocytopenia. This needs to be further confirmed in future studies.

It is also worth mentioning that, the patient did not respond well to adalimumab. In recent years, some studies have found that pharmacogenetics can predict individual treatment response and adverse events. Scholars have studied the polymorphism of TNF and TNF receptor, FCγ and variants of HLA gene. These can affect the outcome of anti-TNF-α drugs treatment. For example, patients with psoriasis and psoriatic arthritis who carry the SNP + 489AA phenotype may be more likely to respond to adalimumab than to etanercept and infliximab (Murdaca et al., 2014a; Murdaca et al., 2014b). In this example, pharmacogenomics could assist us in identifying more effective medications for patients. Due to the limited scope of existing pharmacogenomics studies, the effect of environmental factors on genetic backgrounds, and the interaction of candidate genes with other loci, a great deal of research is still required in this area.

## Conclusion

We report a patient who developed severe thrombocytopenia after adalimumab treatment. Significant thrombocytopenia associated with anti-TNF-α drugs is very rare. Unlike the other cases, we tried multiple treatment options. The thrombocytopenia reported in our case was more persistent. The platelet count quickly returned to normal after we started IVIG. The pathogenesis of thrombocytopenia and its therapeutic mechanism are still unclear. This may be closely related to the production of ADA in the blood. Our case also confirms that immunoglobulins may be considered for the treatment of refractory thrombocytopenia.

## Data Availability

The original contributions presented in the study are included in the article/supplementary material, further inquiries can be directed to the corresponding author.

## Abbreviations

RA, rheumatoid arthritis; TNF-α, tumor necrosis factor α; MTX, methotrexate; IL-11, interleukin-11; rh-TPO, recombinant human thrombopoietin; IVIG, intravenous immune globulin; eGFR, estimated glomerular filtration rate; ITP, immune thrombocytopenic purpura; TTP, thrombotic thrombocytopenic purpura; AA, aplastic anemia; DMARDs, disease-modifying anti-rheumatic drugs; ADA, anti-drug antibodies; DEPP, double filtration plasmapheresis; RTX, rituximab
